# Effects of time-of-day on the noradrenaline, adrenaline, cortisol and blood lipidome response to an ice bath

**DOI:** 10.1038/s41598-025-85304-8

**Published:** 2025-01-08

**Authors:** Alexander Braunsperger, Maximilian Bauer, Chaima Ben Brahim, Lea Seep, Dominik Tischer, Mirko Peitzsch, Jan Hasenauer, Sieglinde Hechenbichler Figueroa, Anna Worthmann, Joerg Heeren, Kenneth A. Dyar, Karsten Koehler, Ana Soriano-Arroquia, Martin Schönfelder, Henning Wackerhage

**Affiliations:** 1 Professorship of Exercise Biology, Department Health and Sport Sciences, TUM School of Medicine and Health, Technical University of Munich, Munich, Germany; 2 Computational Biology, Life & Medical Sciences (LIMES) Institute, University of Bonn, Bonn, Germany; 3 Institute for Pharmacology and Toxicology, Biomedical Center, University of Bonn, Bonn, Germany; 4 Institute for Clinical Chemistry and Laboratory Medicine, University Hospital and Medical Faculty Carl Gustav Carus, TU Dresden, Dresden, Germany; 5 Helmholtz Center Munich, German Research Center for Environmental Health, Computational Health Center, Munich, Germany; 6 Professorship of Exercise, Nutrition and Health, Department Health and Sport Sciences, TUM School of Medicine and Health, Technical University of Munich, Munich, Germany; 7 Department of Biochemistry and Molecular Cell Biology, University Medical Center Hamburg-Eppendorf, Hamburg, Germany; 8 Metabolic Physiology, Institute for Diabetes and Cancer, Helmholtz Diabetes Center, Helmholtz Munich, German Research Center for Environmental Health, Neuherberg, Germany; 9 German Center for Diabetes Research (DZD), Neuherberg, Germany

**Keywords:** Ice bath, Cold exposure, Lipid metabolism, Circadian rhythm, Lipolysis, Noradrenaline, Endocrinology, Lipids

## Abstract

While the effect of time-of-day (morning versus evening) on hormones, lipids and lipolysis has been studied in relation to meals and exercise, there are no studies that have investigated the effects of time-of-day on ice bath induced hormone and lipidome responses. In this crossover-designed study, a group of six women and six men, 26 ± 5 years old, 176 ± 7 cm tall, weighing 75 ± 10 kg, and a BMI of 23 ± 2 kg/m^2 ^had an ice bath (8–12 °C for 5 min) both in the morning and evening on separate days. Absence from intense physical exercise, nutrient intake and meal order was standardized in the 24 h prior the ice baths to account for confounders such as diet or exercise. We collected venous blood samples before and after (5 min and 30 min) the ice baths to measure hormones (noradrenaline, adrenaline, and cortisol) and lipid levels in plasma via liquid chromatography mass spectrometry shotgun lipidomics. We found that ice baths in the morning increase plasma fatty acids more than in the evening. Overall plasma lipid composition significantly differed in-between the morning and evening, and only in the morning ice bathing is accompanied by significantly increased plasma fatty acids from 5.1 ± 2.2% to 6.0 ± 2.4% (*P* = 0.029) 5 min after and to 6.3 ± 3.1% *(P* = 0.008) 30 min after. Noradrenaline was not affected by time-of-day and increased significantly immediately after the ice baths in the morning by 127 ± 2% (pre: 395 ± 158 pg/ml, post 5 min: 896 ± 562 pg/ml, *P* = 0.025) and in the evening by 144 ± 2% (pre: 385 ± 146 pg/ml, post 5 min: 937 ± 547 pg/ml, *P* = 0.015). Cortisol was generally higher in the morning than in the evening (pre: 179 ± 108 pg/ml versus 91 ± 59 pg/ml, *P* = 0.013; post 5 min: 222 ± 96 pg/ml versus 101 ± 52 pg/ml, *P* = 0.001; post 30 min: 190 ± 96 pg/ml versus 98 ± 54 pg/ml, *P* = 0.009). There was no difference in the hormonal and lipidome response to an ice bath between women and men. The main finding of the study was that noradrenaline, adrenaline, cortisol and plasma lipidome responses are similar after an ice bath in the morning and evening. However, ice baths in the morning increase plasma fatty acids more than in the evening.

**Supplementary Information:**

The online version contains supplementary material available at 10.1038/s41598-025-85304-8.

## Introduction

Humans use several forms of cold exposure such as cold showers, Kneipp baths, ice or winter baths, cryotherapy or cold air exposure to achieve several outcomes, including:

1. Increased energy expenditure through the stimulation of thermogenesis^1–3^ especially because of brown adipose tissue (BAT) activation^4,5^,
2. Tumour suppression^6^,
3. Improved immune function^7,8^,
4. Prevention of cardiometabolic diseases^9^,
5. Increase of red blood cell counts and erythropoietin levels^10,11^.
6. Improved circulation^12,13^,
7. Muscular recovery and pain relief in sports^14–16^,
8. Improvement of mood and mental health^16,17^.

However, evidence for these effects may also be attributed to variations in study design, including factors such as temperature and differing types of cold exposure. Other reports claim voluntary meditation regulates the physiological response to cold exposure^18,19^. Further, humans respond differently to interventions such as exercise or cold baths. For example, adjusted VO_2_max adaptation to 20 weeks of endurance training can range from 0.1 to 1 l/min^20^. Factors contributing to this variability include genetics^20,21^, age^22,23^, and sex^24,25^, among others. So far, sex differences in the response to cold exposure have not been investigated.

Recently researchers have investigated on how time-of-day and associated circadian rhythms can affect responses to interventions. For example, several observational studies investigating responses to meals and exercise performed at different times of the day have captured major functional (i.e. exercise capacity and performance), metabolic, hormonal and transcriptional differences, depending on when the intervention occurred^24,26–29^. These differences are largely thought to reflect differences in nutritional state^26,30–32^ along with local tissue and systemic metabolic programs controlled by the circadian timing system. In particular, time of feeding and duration of fasting is known to influence intra- and extracellular signalling^33^. Also, seasonal changes in light can affect energy metabolism by altering hormonal, behavioural and tissue-specific gene expression rhythms^34^. Metabolism and circadian clock gene expression are closely connected since exercise stimuli positively affect the molecular clock of tissues and cells^35^, while clock disruption (e.g. jet lag) or chronic misalignment (e.g. shift work and late chronotypes) promotes insulin resistance and increases risk for cardiometabolic diseases^36,37^. Timing of exercise can set the clock by affecting the magnitude and type of metabolites impacted by exercise^28^, often in a tissue-specific manner. Altered 24-hour metabolite dynamics within and across different tissues in response to diet, exercise, and tissue-specific circadian disruption suggests inter-organ crosstalk (e.g. muscle-fat crosstalk) may likewise be under circadian control^38–41^.

Time-of-day effects on metabolism have mainly been studied in relation to meals and exercise. So far, no studies have investigated these effects after an ice bath in humans, which presumably modulates similar pathways as diet and exercise^42^. Known physiological responses to cold-water immersion comprise, e.g. an increase in plasma noradrenaline^43^ and decrease in plasma cortisol^16^. Also, application of a cold stimulus increases whole-body thermogenesis and the breakdown of stored fatty acids in mice^12^, however this has not yet been demonstrated in humans. Further, it is unclear if circadian rhythms alter the blood lipid response to an ice bath. Cold exposure might not only breakdown lipids in blood, but also in tissue. Namely, adipocyte thermogenesis is enhanced via adrenergic stimulation (e.g. by noradrenaline or adrenaline) leading to increased cardiovascular responses and white adipose tissue lipolysis^44^. Notably, the expression of adrenergic receptors and lipolytic activity of adipocytes is affected by time-of-day as well as the lipid metabolism of mice in response to exercise^38^. Apart from the tissue response to cold-induced hormonal stimuli, the levels of some hormones are known to be following a diurnal rhythm across 24 h. For example we already know that salivary cortisol levels follow a diurnal rhythm and peak in the morning^45^. In contrast, plasma noradrenaline and adrenaline levels peak during the night phase^46^, but little is known on the diurnal response of these hormones in response to cold-water immersion. However, the role of skeletal muscle should not be neglected. Skeletal muscle reveals a circadian pattern in substrate utilization, preferentially relying more on carbohydrate oxidation in the morning and more lipid oxidation in the evening^29^. Further, exercise has been discussed as a potential chronotherapeutic strategy, because of its time-of-day effects on gene expression of skeletal muscle^47^. Therefore, skeletal muscle could potentially affect the response to cold exposure by muscle shivering, which has been shown to increase serum fatty acids after one hour of shivering^48^. Muscle shivering upon cold exposure can be assessed by, e.g. measuring blood lactate since lactate is a byproduct of skeletal muscle glycogen and glucose metabolism^49^. Lactate acts as a three-carbon source for glycolytic ATP production to generate heat via uncoupled mitochondrial respiration^50^.

To fill the discussed gaps in our knowledge, we designed an exploratory human physiology experiment^51^ to test the hypothesis that time-of-day affects the blood levels of noradrenaline, adrenaline, cortisol and the blood lipidome at rest and after an ice bath. The aim of our study was to generate experimental data to answer the following three questions:

1. Do blood hormone concentrations differ in response to a 5-minute ice bath in the morning or evening?
2. Can we observe differences between women and men with the limitation of a small sample size?
3. Do blood lipid class composition and lipid concentrations differ in response to a 5-minute ice bath in the morning or evening?

## Materials and methods

### Ethics

The study was approved by the Ethics Committee of the Technical University of Munich and performed in accordance with the Declaration of Helsinki. The registration number of the study is “2023-249-S-KH”.

### Eligibility, informed consent and baseline assessments

After recruitment, the participants were invited to the Prevention Center of the Technical University of Munich for physical examination. The PAR-Q + form as well as a questionnaire to determine the inclusion/exclusion criteria was completed^52^. Also, signed informed consent for both the study participation as well as the publication of all the collected information in an open access publication was obtained. During the initial physical examination, the inclusion/exclusion criteria were verified during an interview with a trained study physician. Inclusion criteria was a BMI between 20–25 kg/m^2^ and 18–40 years of age. Predetermined exclusion criteria were chronic cardiovascular disease, respiratory, neoplastic, or metabolic conditions (e.g. lipid disorder) and pregnancy. Resting ECG was recorded in a supine position to check possible heart valve defects, which would lead to withdrawal from study participation. Also, resting blood pressure measurements were conducted and had to be below 120/80 mmHg. Further we noted any self-reported allergies, injury history and current intake of any medications. No participant revealed an intake of ADHD medications, which could affect noradrenaline and adrenaline measurements^53^. Any possible hint of drug abuse or excessive alcohol intake led to elimination from the study. In addition, the participants were informed about potential risks of ice bathing such as hypothermia and unconsciousness due to limited breathing. Finally, they received a structured procedure on how the study would be conducted.

### Familiarization

After successful inclusion, two familiarization sessions were scheduled leading up to the first ice bath experiment. The participants had to take a 3-minute ice bath (12–14 °C) twice to familiarize to the cold for the intervention. The ice bath during familiarization had a higher water temperature (12–14 °C) than the ice bath during the experiments (8–12 °C) to ensure acclimation to the cold-water while not introducing a training effect. Past studies showed that cold-water acclimation protocols can increase comfort during cold-water immersion and reduce muscle shivering intensity^54,55^. During the first ice bath sessions, participants received guided breathing technique training to reduce the risk of hyperventilation and to avoid adverse responses possibly leading to the abortion of the test. The instruction for the breathing technique was to purposefully exhale and the time of exhalation should exceed the time of inhalation as this way of breathing may prevent from hyperventilation^56^. One week was scheduled between familiarization and the first ice bath experiment to avoid adaptation effects. All measurements of the intervention were scheduled during a 2-month period from September until October.

### Experimental design

**Fig. 1:**
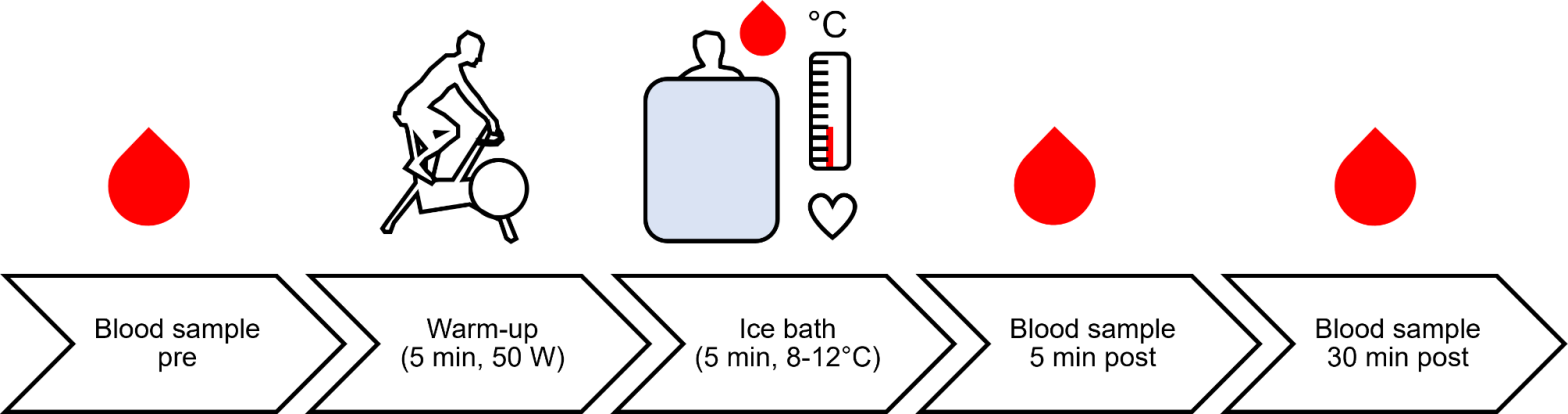
Experimental design of the ice bath experiments in the morning and evening. The red blood dots represent venous blood sampling timepoints (pre, 5 min post and 30 min post). Participants conducted a 5-minute warm-up on a cycle ergometer at 50 Watts before the ice bath to increase the heart rate before and avoid an adverse response after cold-water immersion. During the ice bath we measured water temperature (at the start and end of the ice bath) and the participants heart rate (at rest, after each minute in the ice bath and 5 min post). Further, we sampled capillary blood from the earlobe to measure blood lactate after the warm-up before the ice bath, 2 and 4 min into the ice bath as well as immediately and 5 min after the ice bath.

Participants were instructed to not engage in any intense physical activities or exercise and to not drink any alcohol or caffeine 24 h prior to the measurements. Adherence to these instructions was controlled by a self-report of the participants on the day of the experiment. To account for circadian differences in the lipidome response to an ice bath, the participants had to wake up at the same time (5:30 AM) on each day of an ice bath. Sleeping times and hours had to be documented and were sent to us to ensure the participants had 7–8 h of sleep. The participants were assigned to do both a morning ice bath (8:00–10:00 AM) and evening ice bath (5:00–7:00 PM) depending on their weekly availabilities. There was at least one week scheduled between the two ice bath measurements to avoid interfering effects. Each day of an ice bath experiment followed the same standardized protocol (Fig. 1). To address possible nutritional interferences, the participants were instructed to follow our standardized nutritional guidelines. The final two meals before each ice bath were standardized and consumed within 12 h before each ice bath in the same order. A standardized meal (10.4 kcal per kg bodyweight: 2 g carbohydrates, 0.4 g proteins, and 0.2 g fats per kg bodyweight) and a standardized snack (9.8 kcal per kg bodyweight: 1.5 g carbohydrates, 0.5 g proteins, and 0.2 g fats per kg bodyweight) were consumed before each ice bath. Therefore, participants consumed a total of 20.2 kcal per kg of their bodyweight in the 12 h before either morning or evening ice bath. Some of the participants were vegetarians hence we created vegetarian meals (meal: egg noodles with tofu & hoisin sauce; breakfast: greek yoghurt with oats & honey). Macro nutrients were prescribed per kg of bodyweight based on the guidelines from the German Nutrition Society „Richtwerte für die Energiezufuhr aus Kohlenhydraten und Fett. *Deutsche Gesellschaft für Ernährung.*“ (https://www.dge.de//fileadmin/dok/wissenschaft/positionen/DGE-Positionspapier-Richtwerte-Energiezufuhr-KH-und-Fett.pdf) (2011). The standardized meal had to be eaten 12 h before the morning ice bath (as dinner on the night before) or 5 h before the evening ice bath (as lunch). The standardized snack had to be consumed two hours before both ice baths, but not together with the meal. To check adherence, the participants reported the timing of both meals and reported to us. Every participant finished the snack 2 h before each of the ice baths. A physician collected a venous blood sample at rest before the 5-minute ice bath. Then, a short 5-minute warm-up (50 Watts) on a stationary cycle ergometer was performed to elevate heart rate prior to the cold-water immersion and avoid an adverse reaction due to the cold. The participants immersed into the cold water (8–12 °C) up to their armpits and left one arm out of the tub for venous blood sampling afterwards. Once the participants immersed in the cold water, they were instructed to follow the familiarized breathing technique. Water temperature was continuously monitored and crushed ice was added to the water to avoid an increase of water temperature. Then, 5 and 30 min after the 5-minute ice bath, further venous blood samples were collected to investigate the time course response of our readouts. A duration of 5 min was chosen for the ice baths since this seems to be a commonly used duration of athletes for recovery after exercise^57–59^ or during recreational ice bathing during the winter months.

### Blood sampling

The venous blood samples were collected in EDTA monovettes, gently inverted four times and immediately centrifuged (room temperature, 2.500 G, 10 min). Blood plasma was aliquoted, and stored at -80 °C. We sent the samples on dry-ice to the University Medical Center Hamburg-Eppendorf for lipidomic analysis and to the University Hospital Carl Gustav Carus in Dresden for the measurement of catecholamines. Samples were labeled anonymously and tested in random order. Capillary blood was sampled from the earlobe using safety lancets and capillaries of a volume of 20 µl, which we put in a tube filled with hemolysis buffer. Blood lactate levels were then measured on a lactate analyzer (EKF Diagnostic; Biosen S-line).

### Catecholamine analysis

Noradrenaline and adrenaline were measured by liquid chromatography with electrochemical detection (LC-ECD) as described elsewhere^60^. Cortisol levels were quantified by an enzyme immunoassay (Cortisol ELISA, DRG, EIA-1887, sensitivity: 2.5 ng/ml, CV%: 6.5–7.7%, measuring unit: ng/ml).

### Lipidomic analysis

Shotgun lipidomic analysis of plasma samples was performed as described by Su et al.^61^ and lipids were extracted from plasma employing an adjusted MTBE/methanol extraction protocol^62^. Briefly, plasma samples were spiked with internal standards and subsequently mixed with MTBE/methanol/water 10:3:2.5 (v/v/v). Following homogenization, samples were centrifuged (4 °C, 10.000 G, 10 min) and the lipid containing upper phase was transferred into a new vial. Lipid extracts were concentrated by vacuum centrifugation and reconstituted in a mixture of dichloromethane (50): methanol (50) containing 10 mM ammonium acetate. Direct infusion of lipid extracts into the mass spectrometer was conducted by an ultrahigh-pressure liquid chromatography system (Nexera X2, Shimadzu, Kyoto, Japan) using dichloromethane (50): methanol (50) containing 10 mM ammonium acetate as eluent at a flowrate of 0.008 ml/min^61^. The separation and targeted profiling of lipid species was performed combining differential mobility spectrometry and a QTRAP^®^ system (QTRAP^®^ 5500, SCIEX) run in multiple reaction monitoring mode operated via Analyst (version 1.6.8, SCIEX). The mass spectrometry generated raw data were converted to mzML format using MSconvertGUI (Version 3.0.21245-5724be1). Data processing and lipid quantification were performed using the Shotgun Lipidomic Assistant (SLA) software, a python-based application according to Su et al.^61^.

### Statistics

#### Catecholamines

The catecholamine data was statistically analysed to investigate time-of-day and sex differences with repeated measures two-way analysis of variance (ANOVA). A general linear model was used if there were no missing values and a mixed effects model with maximum likelihood method if there were missing values. Fisher’s least significant difference test was used to make multiple comparisons and Greenhouse-Geisser correction to adjust for lack of sphericity.

#### Lipidomics

A total of 945 species were detected across 12 participants, with one participant and its four associated measurement timepoints excluded due to unsuccessful venous blood sampling after the ice bath. To eliminate the need for imputation, only lipids from measurements without missing values were included. The final set consisted of 305 lipids measured over 64 plasma samples. The data of the lipid concentrations was log_2_ transformed, to achieve an approximate normal distribution. The resulting normal distribution was validated by using QQ-plots (Supplementary material 1). This resulting dataset was subjected to principal component analysis (PCA). PCA transforms the data into new variables called principal components (PCs), which are ranked based on how much information (variance) they capture from the original dataset. By projecting the data onto the first two components – capturing 32.8% and 17.6% variance, respectively – we reduce the dimensionality from 305 to two, but retain a significant part of the original data’s variation. Samples were color-coded based on their fatty acid (FA) composition to assess trends along the axes of greatest variation. Time-of-day information (morning or evening) was also annotated on the PCA plots through differing shapes. Additionally, measurements from individual participants were connected in PCA space to highlight intra-individual variability, with areas formed by these connections used as surrogates for intra-individual variance. This facilitates a visual inspection of the dataset. The code to reproduce the PCA analysis’ figure can be retrieved from: 10.5281/zenodo.13322184. The blood lipid composition data was statistically analysed to investigate time-of-day and sex differences with repeated measures two-way ANOVA. A general linear model was used if there were no missing values and a mixed effects model with maximum likelihood method if there were missing values. Fisher’s least significant difference test was used to make multiple comparisons and Greenhouse-Geisser correction to adjust for lack of sphericity. For the statistical analysis of the plasma lipid concentrations, the *z*-scores for each lipid concentration were calculated using the formula: *Z* = $\:\frac{(x\:-\mu\:)}{\sigma\:}$. Two-tailed t-tests were computed between all measurement timepoints for the plasma fatty acid concentrations (Z-scores). Pearson’s R was computed to investigate the relationship of any increased catecholamine data in response to the ice bath with changes in blood lipid concentrations.

## Results

### Monitoring of water temperature and heart rate during the ice baths

**Fig. 2:**
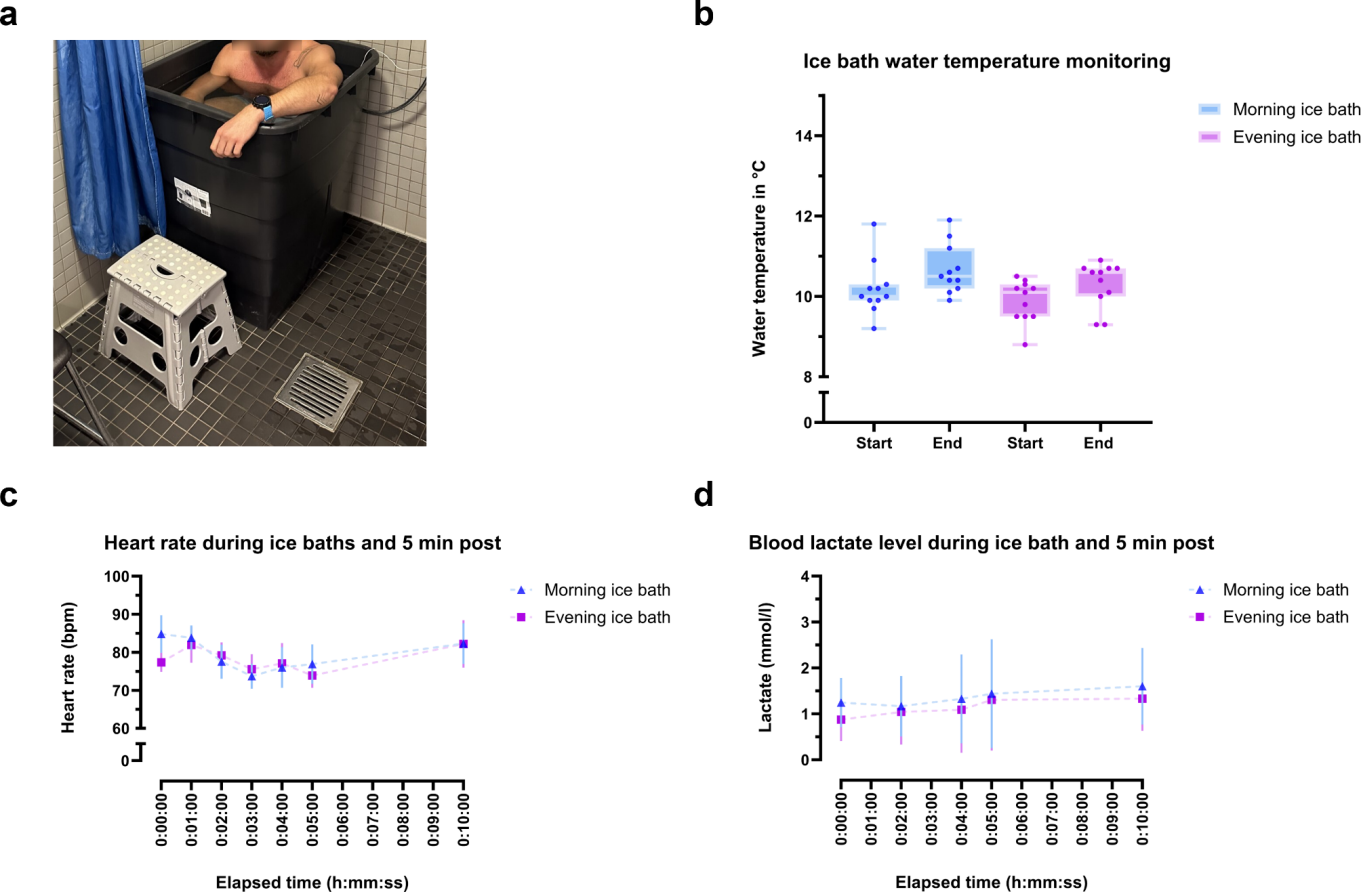
Ice bath set-up and monitoring. (**a**) Ice bath experimental set-up. (**b**) Monitoring of the 5-minute ice bath temperature at the start (water temperature after participant fully immersed into the cold water) and at the end (water temperature at the end of the 5th minute of the ice bath). Error bars indicate minimum and maximum measured water temperature. (**c**) Monitoring of heart rate at rest (0:00:00), after each minute of the ice bath (0:01:00 to 0:05:00) and 5 min post (0:10:00). Error bars indicate standard deviation. (**d**) Monitoring of capillary blood lactate levels before (0:00:00), after two and four minutes of the ice bath (0:02:00 to 0:04:00) and 5 min post (0:10:00). Error bars indicate standard deviation.

To compare the effects of a 5-minute, 8–12 °C ice bath on hormone and lipid concentrations in the morning (8:00–10:00 AM) versus the evening (5:00–7:00 PM) we randomly assigned 12 volunteers (women and men) to take ice baths at both times. The included six women and six men were 26 ± 5 years old, 176 ± 7 cm tall, weighed 75 ± 10 kg, and had a BMI of 23 ± 2 kg/m^2^. Supplementary material 2 lines up participants characteristics of both women and men. Once the participants immersed in the cold water (Fig. 2a), water temperature was measured (Fig. 2b) as well as at the end of the 5th minute of the ice bath. During the 5-minute ice bath, ice cubes were put into the water to avoid an increase of the water temperature as much as possible. Heart rate was recorded via wristwatch and is reported in Fig. 2c at rest, during the 5-minute cold-water immersion and 5 min post immersion. Heart rate did not change significantly during the 5-minutes in cold water or 5 min post. Figure 2d shows monitoring of blood lactate levels, as a possible indication for skeletal muscle shivering, did not change significantly from before, to during and after the ice bath.

### The plasma hormone response to the ice bath is not influenced by time-of-day or by sex

**Fig. 3:**
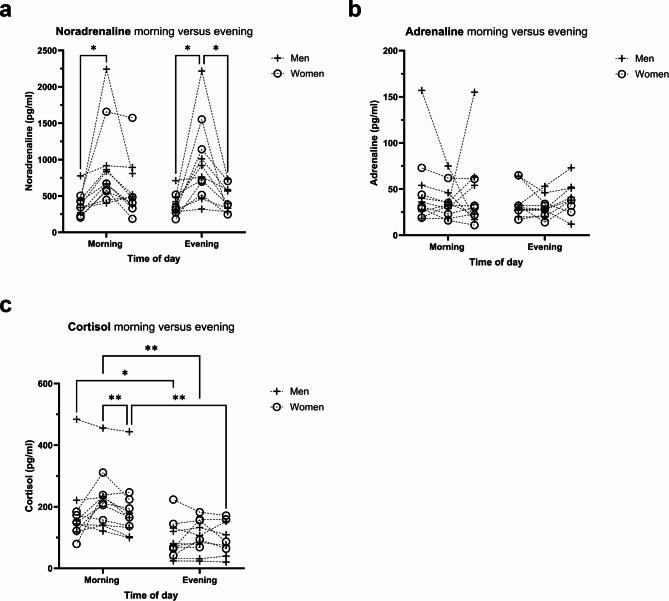
Hormonal response to the ice baths. Plasma concentrations of (**a**) noradrenaline, (**b**) adrenaline and (**c**) cortisol from before to 5 and 30 min after an ice bath. Noradrenaline increases significantly 5 min after an ice bath, irrespective of the time-of-day. Adrenaline and cortisol are not affected significantly by any ice bath. Two adverse responses in plasma adrenaline concentrations of participants can be observed. Plasma cortisol concentrations are significantly higher in the morning than in the evening. Two-way ANOVA. (*P < 0.05; **P < 0.01). Non-shown comparisons were statistically not significant.

We measured the plasma concentrations of noradrenaline, adrenaline and cortisol before as well as 5 and 30 min after the ice baths (Fig. 3). After pre-post comparison we found that the concentrations of noradrenaline (Fig. 3a) significantly increased by 127 ± 2% from 395 ± 158 pg/ml to 896 ± 562 pg/ml (*P* = 0.025) 5 min after the morning ice bath and by 144 ± 2% from 385 ± 146 pg/ml to 937 ± 547 pg/ml (*P* = 0.015) 5 min after the evening ice bath. Noradrenaline levels dropped back down to 564 ± 382 pg/ml in the 30 min after the morning ice bath and back down to 448 ± 185 pg/ml (*P* = 0.022) in the 30 min after the evening ice bath. There was no significant difference between morning and evening timepoints. In contrast, adrenaline varied only in two volunteers in the morning (Fig. 3b). Adrenaline did not change significantly in response to the ice bath in the morning (pre: 48 ± 40 pg/ml; post 5 min: 37 ± 18 pg/ml; post 30 min: 44 ± 41 pg/ml) nor in the evening (pre: 34 ± 16 pg/ml; post 5 min: 30 ± 11 pg/ml; post 30 min: 40 ± 17 pg/ml). Though, this represents a mean percentage change of 23 ± 1% and − 11 ± 1% 5 min after and − 8 ± 2% and 18 ± 1% 30 min after the morning and evening ice bath, respectively. The concentration of cortisol (Fig. 3c) was generally higher in the morning than in the evening (pre: 179 ± 108 pg/ml versus 91 ± 59 pg/ml, *P* = 0.013; post 5 min: 222 ± 96 pg/ml versus 101 ± 52 pg/ml, *P* = 0.001; post 30 min: 190 ± 96 pg/ml versus 98 ± 54 pg/ml, *P* = 0.009), but did not respond significantly to the ice bath at either timepoint except in the morning from 5 min post ice bath to 30 min post from 222 ± 96 pg/ml to 190 ± 96 pg/ml (*P* = 0.010). However, the cortisol response was not significantly impacted by the ice baths, a mean percentage increase of 24 ± 1% and 11 ± 1% 5 min after as well as 6 ± 1% and 7 ± 1% 30 min after morning and evening ice bath can be observed, respectively. Overall, we observed no significant differences in responses between women and men.

### The time-of-day affects plasma lipid class composition, and morning ice baths increase the relative number of plasma fatty acids

**Fig. 4:**
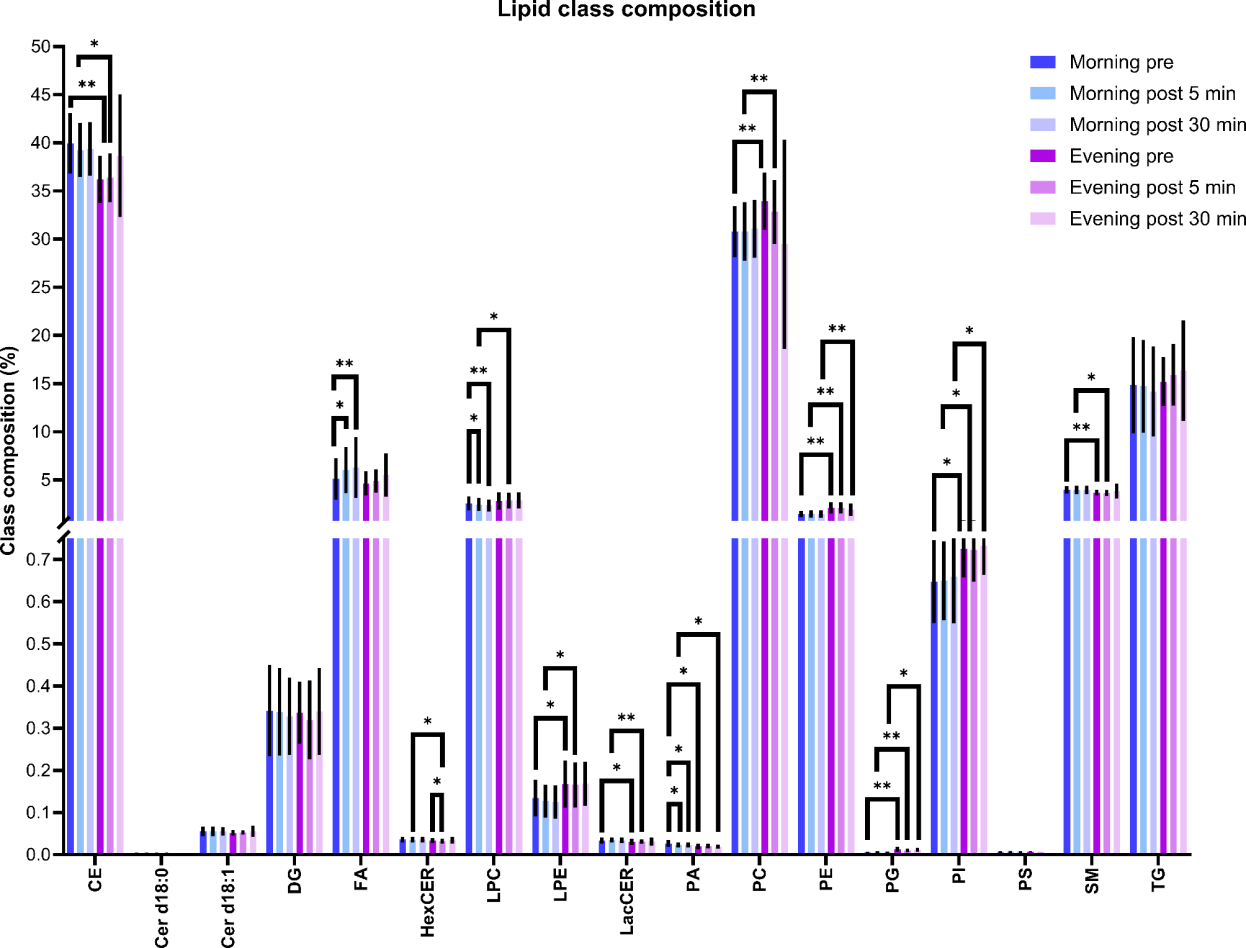
Mean lipid class composition (%) of the measured 64 plasma samples. CE: cholesteryl esters; Cer: ceramides; DG: diacylglycerols; FA: fatty acids; HexCER: hexosylceramides; LPC: lyso glycerophosphocholines; LPE: lyso glycerophosphoethanolamines; LacCER: lactosylceramides; PA: glycerophosphates; PC: glycerophosphocholines; PG: glycerophosphoglycerols; PI: glycerophosphoinositols; PS: glycerophosphoserines; SM: sphingomyelins; TG: triacylglycerols. Most of the lipid classes significantly differ between morning and evening. FA, PA and LPC significantly differ from pre to post the morning ice bath. Two-tailed paired sample t-test. (*P < 0.05; **P < 0.01). Non-shown comparisons were statistically not significant. Error bars show standard deviation.

One of the functions of noradrenaline is to stimulate lipolysis, which is the breakdown of triacylglycerol (i.e. fat) into glycerol and free fatty acids. Since we observe a clear response of noradrenaline following an ice bath, we further investigated the blood lipid response of lipids to this stimulus. Despite the rise of noradrenaline, the mean relative lipid class composition changed little in response to an ice bath but differed in-between morning and evening (Fig. 4). Most differences can be found between morning and evening timepoints and less, but some across the timepoints of the morning measurements: fatty acids (FA), lyso glycerophosphocholines (LPC), and glycerophosphates (PA). Interestingly, the relative number of fatty acids in the plasma significantly increased from 5.1 ± 2.2% before the morning ice bath to 6.0 ± 2.4% 5 min post (*P* = 0.029) and to 6.3 ± 3.1% 30 min post (*P* = 0.009). Lyso glycerophosphocholines (LPC) and glycerophosphates (PA) decrease was statistically significant but represents a relative change of less than 1% of absolute values close to zero. These lipid classes were left out from further interpretation.

### The increase of plasma fatty acid concentrations is consistent with increased noradrenaline levels in response to the ice bath

**Fig. 5:**
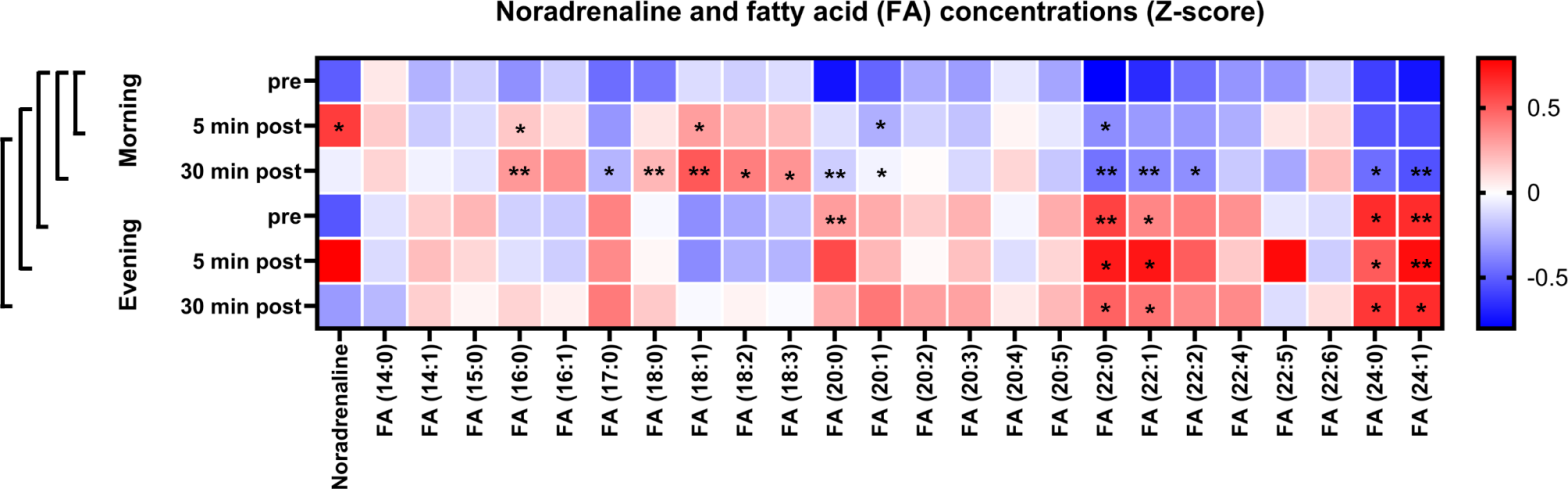
Heatmap of plasma noradrenaline and plasma fatty acid concentrations (Z-scores) for each timepoint. Morning versus evening comparisons reveal significant differences of specific fatty acids. Before the evening ice bath, the plasma fatty acid concentrations are higher compared to before the morning ice bath. Plasma fatty acid concentrations increase following the morning ice bath and this is consistent with the increase of plasma noradrenaline 5 min post. The brackets on the left of the heatmap visualize the illustrated t-test comparison (*P < 0.05; **P < 0.01). Two-tailed t-tests.

**Fig. 6:**
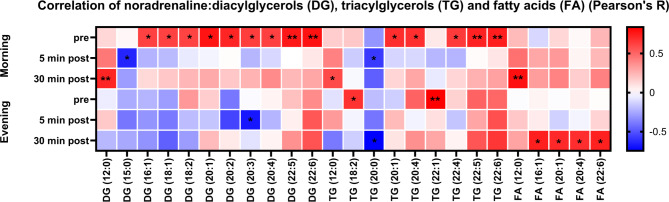
Correlation of plasma noradrenaline and plasma lipid concentrations. DG: diacylglycerols; TG: triacylglycerols; FA: fatty acids. In response to the morning ice bath a pattern switch from positive to no correlation or few negative correlations between plasma noradrenaline and DG as well as TG concentrations can be observed. Some specific fatty acid concentrations reveal a pattern switch towards a positive correlation 30 min post both after a morning and evening ice bath. Only the lipids with significant correlations are shown (*P < 0.05; **P < 0.01). Pearson’s R.

Due to the detected increase of the relative number of fatty acids in the plasma, we computed the z-scores to investigate possible changes of the fatty acids’ concentrations (Fig. 5). Thereby, we observed significantly higher fatty acid z-scores before the ice bath in the evening compared to morning (FA (22:0), FA (22:1), FA (24:0), FA (24:1)). Also, we found significantly increasing fatty acid Z-scores from before to both 5 min post morning ice bath (FA (16:0), FA (18:1), FA (20:1), FA (22:0)) and 30 min post morning ice bath (FA (16:0), FA (17:0), FA (18:1), FA (18:2), FA (18:3), FA (20:0), FA (20:1), FA (22:0), FA (22:1), FA (22:2), FA (24:0), FA (24:1)). We calculated the z-score of noradrenaline at each timepoint and observed that the increase in noradrenaline is only consistent with the plasma fatty acid response in the morning. To further elucidate noradrenaline-associated patterns and the effect on lipid catabolism we computed the correlation of noradrenaline with diacylglycerols (DG), triacylglycerols (TG) and fatty acids (FA). Several significant correlations appear between noradrenaline and the chosen lipid classes (Fig. 6). At timepoints ‘Morning pre’ and ‘Morning 5 min post’ we observed a pattern switch from positive correlations (e.g. DG and TG) to no correlations or few negative correlations (e.g. DG (15:0) and TG (20:0)). When looking at the fatty acids, positive correlations appear 30 min after the ice bath in the morning (FA (12:0)) and in the evening (e.g. FA (16:1), FA (20:1), FA (20:4), FA (22:6)). After the evening ice bath, we only observed the pattern switch from significant positive correlation to no correlation for TG (18:2) and TG (22:1). The only significant negative correlation in response to the evening ice bath was found 5 min post for DG (20:3) and 30 min post for TG (20:0). Positive correlations between noradrenaline and fatty acids can be observed 30 min post morning ice bath (DG (12:0) and FA (12:0) and 30 min post evening ice bath (FA (16:1), FA (20:1), FA (20:4) and FA (22:6)).

### Plasma fatty acids could explain the most variance caused by the ice baths across all plasma samples and is highly individual

**Fig. 7:**
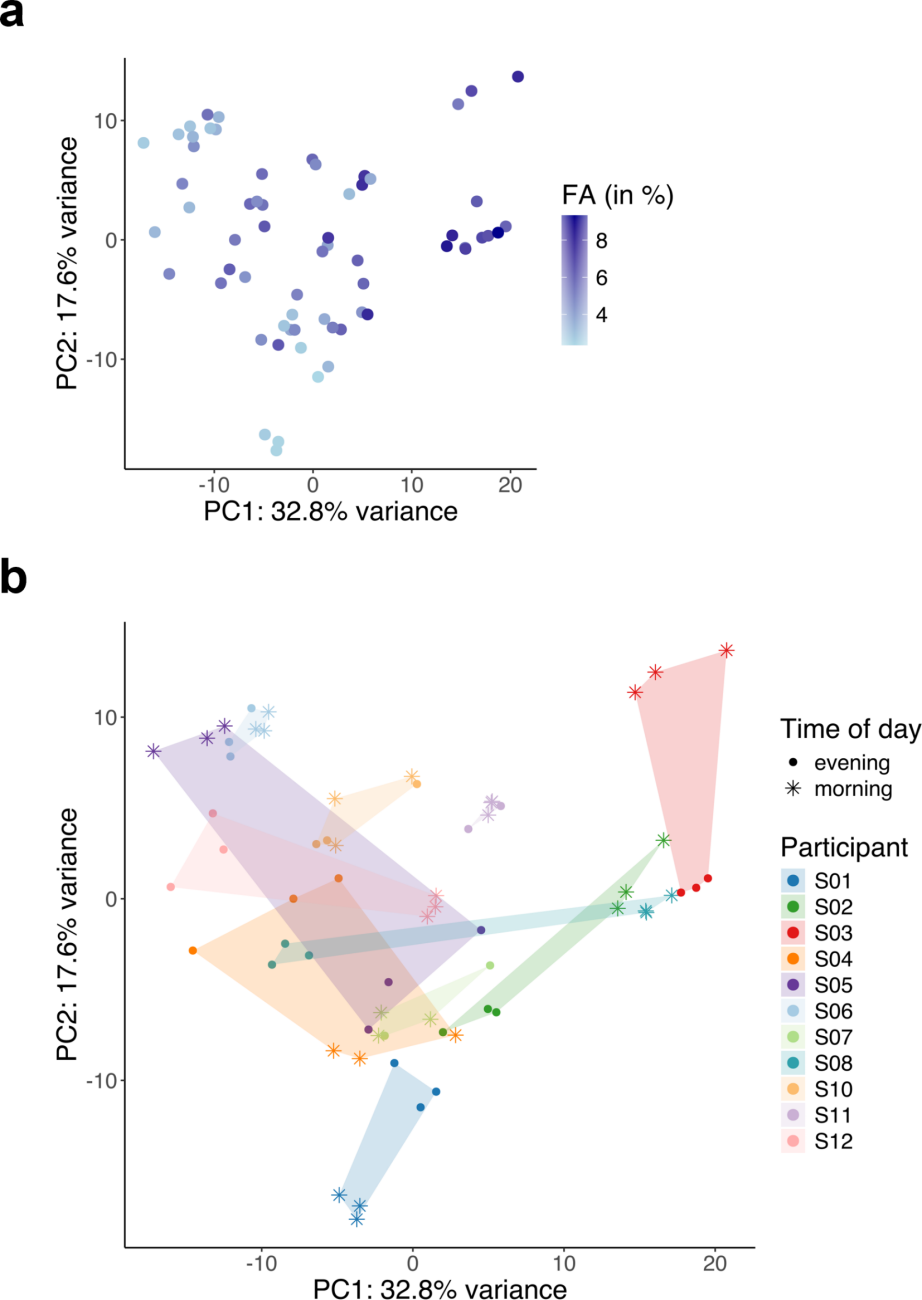
Principal component analysis (PCA) of the plasma lipid concentrations. Principal component (PC) 1 captured 32.8% and PC 2 captured 17.6% of the variance of the individual plasma lipid concentrations across a total of 64 plasma samples measured during Shotgun Lipidomics Analysis. (**a**) The blue color-code indicates the plasma free fatty acid composition (in %) and reveals a clear gradient along the diagonal of the plot. Thus, individual plasma fatty acid composition could explain the most variance in the lipidomic dataset. (**b**) Time-of-day is indicated by symbols and inter-individual variation by color. Most samples show inter-individual time-of-day effects indicated by the separation of symbols of the same color.

**Fig. 8:**
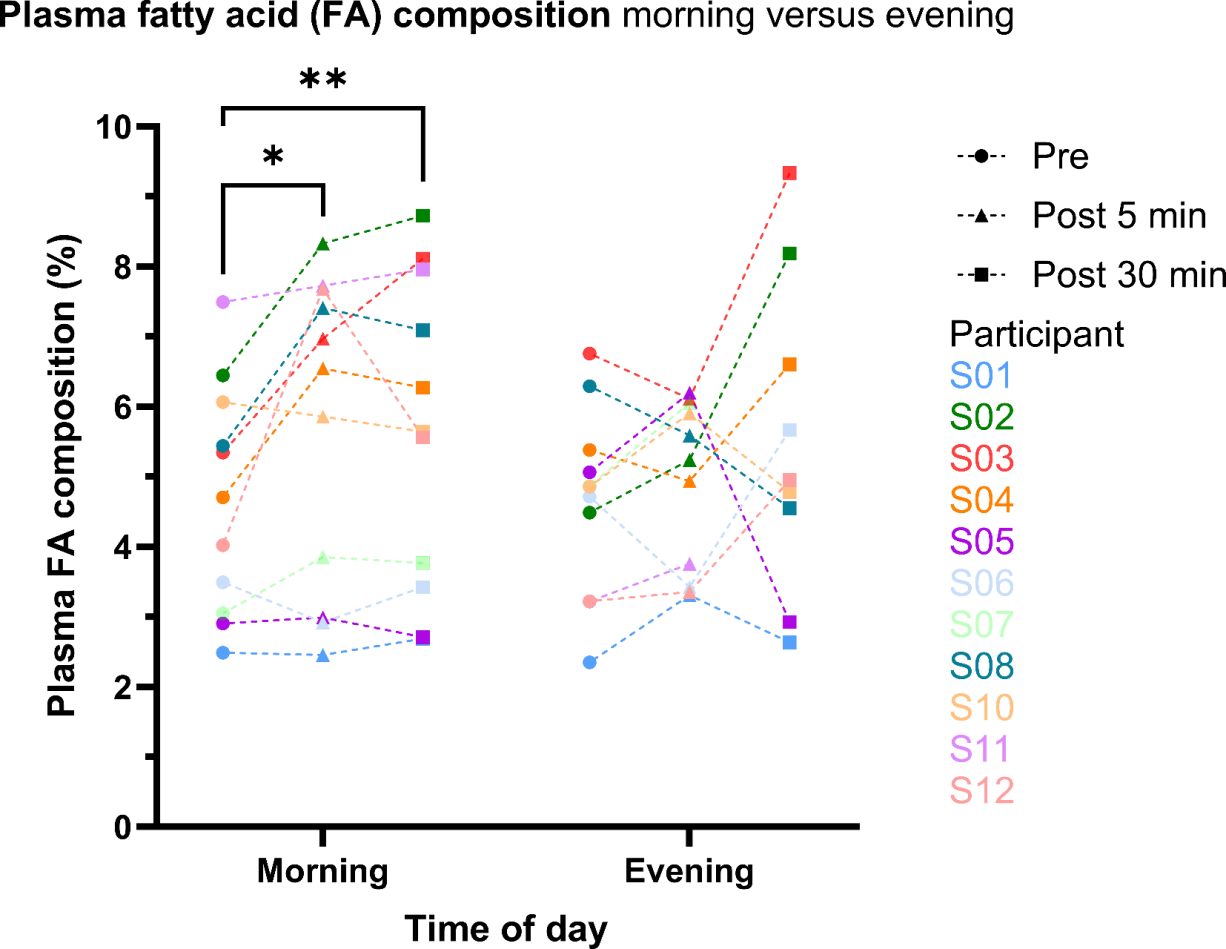
Individual response of plasma fatty acid composition (%) after morning and evening ice bath. Illustrated timepoints are from pre ice bath, 5 min post and 30 min post. 6 participants (S02, S03, S04, S07, S08, S12) show an increase in plasma fatty acid composition 5 min after the morning ice bath. Whereas 5 participants (S01, S05, S06, S010, S11) do not show a response at all. After an evening ice bath there is hardly any or very little response 5 min post and 4 participants (S02, S03, S04, S12) show a response 30 min post. Two-tailed paired sample t-test. (*P < 0.05; **P < 0.01). Non-shown comparisons were statistically not significant.

To investigate the effect of an ice bath on the individual lipidome, we conducted a PCA analysis (Fig. 7a) to evaluate the variance across the measured 64 plasma samples. Principal component (PC) 1 and PC 2 captured 50.4% of the total variance. The blue colour-scale of the data points illustrates the plasma fatty acid composition. The resulting PCA plot revealed a clear gradient in the fatty acid composition along the diagonal of the plot. Notably, while no global clustering patterns were observed with respect to the time-of-day or the timing relative to the ice bath, the majority of samples showed intra-individual time-of-day effects, with the exception of 3 participants: S06, S07, S10 and S11 (Fig. 7b). This indicates that the primary source of variability in the dataset is driven by differences in fatty acid composition rather than time-related factors. Thus, we examined, each individual participant’s plasma fatty acid response after the morning and evening ice bath in more detail (Fig. 8). Since we have not measured any significant differences in the response between women and men, we linked the individual response to an ice bath between the time of day. The majority of the participants showed an increase in the plasma fatty acids 5 min (*P* = 0.029) and 30 min (*P* = 0.009) after the morning ice bath (pre: 5.1 ± 2.2%; post 5 min: 6.0 ± 2.4%; post 30 min ± 3.1%). Some participants revealed no response at all in the morning. After the evening ice bath, the increase in plasma fatty acids is either very small or occurs 30 min post. At last, we checked all our readouts on a possible impact of the order of the ice baths and did not observe any effect.

## Discussion

The aim of this study was to investigate, if hormonal and lipid responses to an ice bath depend on the time-of-day or are affected by sex. Of all the measured hormones, noradrenaline increased the most in response to an ice bath albeit with large inter-individual variation. We observed an increase of plasma fatty acids after an ice bath in the morning, but not in the evening. Cortisol showed higher concentrations in the morning compared to evening. Finally, the greatest source of variation in plasma lipid composition could be explained by plasma fatty acids, which increase in response to the ice bath and this response is highly individual. To our knowledge this is the first experimental human study investigating the effect of time-of-day on plasma hormone and lipidome responses to an ice bath﻿.

Our first aim was to measure, if blood hormones respond differently to a morning and evening ice bath. The hormonal response was affected by both the ice bath and by the time-of-day. In response to the ice baths noradrenaline (Fig. 3a) is increased 2.3-fold in the morning and 2.4-fold in the evening. Therefore, our finding represents a non-pharmacological stimulus releasing noradrenaline into the circulation and possibly might further lead to increased BAT thermogenesis since human brown adipocyte thermogenesis is stimulated by beta-2 adrenergic stimulation^44^. Unexpectedly, while noradrenaline increased after morning and evening ice baths, we only observed increased plasma fatty acids after the morning ice bath. However, in adult humans, supraclavicular BAT can be quantified by ^18^FDG positron emission tomography (PET) scans and reveals that cold-activated human BAT volume is small. Human BAT volume measures between 50 and 150 ml and is probably not the only organ or tissue, e.g. skeletal muscle, contributing to the response on a cold stimulus^63^. Compared to other mild cold exposure experiments on BAT activation, the duration of our ice bath was quite short (5 min versus up to 120 min) and raises the question if this was sufficient to activate BAT in the participants. Likely, the acute response to our ice baths might also be affected by skeletal muscle due to shivering during the ice bath and even possibly by the 5-minute warm-up. Nevertheless, the warm-up intensity was very low, and duration was very short. Figure 2d shows blood lactate levels during the ice bath did not change significantly, but the sampled values are variable. It has been shown that blood lactate increases after prolonged cold-water immersion due to muscle glycogen utilization during shivering^64^. Underlining the possibility of individual differences in muscle shivering during the ice baths, which we also observed in the response of the plasma fatty acid composition (Fig. 8). Notably, exercise is an established synchronizing factor (i.e. Zeitgeber) for skeletal muscle circadian clocks^29,35,65^ and the measured time-of-day effect of the present study on plasma fatty acid composition could also be caused by molecular signaling of shivering skeletal muscle. Recently, prosaposin has been identified as a secreted protein in the extracellular fluid of mouse skeletal muscle and beige adipose tissue. Both, muscle-specific PGC1α overexpression as an exercise-mimicking phenotypic modification and cold exposure led to increased prosaposin expression and secretion in muscle and fat, respectively^66^. This highlights the role of both organs: muscle and fat, secreting myokines and adipokines in response to cold exposure. Besides, we measured no change in adrenaline levels (Fig. 3b). While cortisol concentrations are not affected by an ice bath its concentration levels differ in the morning and evening (Fig. 3c). It is already known that cortisol level peaks in the morning, after fasting overnight, and decreases upon awakening^67,68^. Therefore, our results are in accordance with known physiological rhythms. Notably, the higher cortisol levels in the morning are in line with the increased plasma fatty acids after the ice bath. There is evidence that cortisol and growth hormone can increase lipolytic activity in vitro and in vivo^69–71^.

The second aim was to answer whether there is a difference between women and men. We found no significant sex differences in the response to an ice bath. We acknowledge the limitations associated with a small sample size (6 women and 6 men). Also, we did not control for menstrual cycles. Since both an ice bath and exercise are interventions causing a molecular stimulus in vivo, we could possibly explain this finding by a recent study of the Molecular Transducers of Physical Activity Consortium (MoTrPAC) (Developing the compendium of molecular transducers (the ‘molecular map’) that respond to acute and chronic exercise. *Molecular Transducers of Physical Activity Consortium* https://www.motrpac.org/ (2024)). The MoTrPAC consortium showed that sex differences in response to exercise are organ and tissue specific. Especially, white adipose tissue reveals an upregulation of immune cell activity specific to male mice in adaption to exercise^24^. Thus, our blood analyses might be rather limited to uncover possible sex-dependent effects. Consequently, we argue that future investigations on human cold exposure should collect biopsies from additional tissues, including white and brown adipose tissue as well as skeletal muscle, and expand metabolite and hormone profiling to comprehensively investigate acute effects of an ice bath on a tissue level.

The third aim was to investigate differences in the plasma lipid class composition and the plasma lipid concentrations in response to an ice bath in the morning or evening. The composition of most lipid classes differs in the morning versus the evening. The relative number of plasma fatty acids significantly increases after a morning ice bath from 5% to above 6%, so approximately 20% of itself (Fig. 4). The measured increase in noradrenaline is consistent with higher concentrations of specific fatty acids in plasma after an ice bath in the morning (Fig. 5). In addition, the increase in noradrenaline in response to the evening ice bath was accompanied by a negative correlation with some diacylglycerols (DG) and triacylglycerols (TG) (Fig. 6). Accordingly, we observe an ice bath induced time-of-day effect on our baseline plasma fatty acid concentrations either due to possible activation of BAT thermogenesis, molecular signaling by shivering skeletal muscle or likely both. This resulted in high variation across the plasma samples (Fig. 7b), especially the high variation in plasma fatty acid composition (Fig. 7a). When we looked at the individual plasma fatty acid response to morning and evening ice bath, we found that there is a clear response 5 min after the morning ice bath among 50% of the participants. If there was a response after the evening ice bath, then this occurred 30 min post the ice bath. We interpret our observations in response to an ice bath as noradrenaline-associated lipolysis: breakdown of stored lipids (DG and TG) into fatty acids. In accordance with our study, a lipidomic analysis of fasted human sera after 120 min mild cold exposure showed increased circulating free fatty acids and decreased triglycerides in human sera^12^. Rather, unexpectedly we find that evening ice baths do not increase circulating fatty acids regardless of the increase in plasma noradrenaline in response to the ice bath. Other explanations could be the higher plasma cortisol levels in the morning or else molecular signaling by shivering skeletal muscle during the ice baths since lipolysis is not only regulated by noradrenaline, but several other factors^72^. Further, diet-induced thermogenesis could be a possible confounder, because of the non-matched meal timing in this study. The standardized meal was consumed 12 h before the morning ice bath and 5 h before the evening ice bath. Therefore, the fasting interval between the meal and the snack was shorter before the evening ice bath. Still, each participant consumed the snack exactly 2 h before an ice bath. Diet-induced thermogenesis can increase daily energy expenditure levels during the post absorbative phase, which lasts between 6 and 10 h^73^. The non-matched meal timing could possibly be a reason for the elevated plasma fatty acid concentrations before the evening ice bath compared to before the morning ice bath (Fig. 5). Nevertheless, individual plasma fatty acid composition did not alter before morning or evening ice bath (Fig. 8). Interestingly, the postprandial plasma fatty acid response in humans has been shown to be dependent on time-of-day and is highest in the evening and the lowest after the awakening^74^. Accordingly, a morning ice bath stimulus could possibly cause fatty acid mobilization into plasma when its diurnal rhythm is the slowest. In summary, our findings suggest that morning ice baths increase plasma fatty acid composition, but this effect is less pronounced in response to evening ice baths. Real-life implications of the present study are that 5-minute ice baths after a short and easy 5-minute cycling warm-up in the morning can be used as a chronotherapeutic intervention to increase lipolysis.

### Limitations

This study has the following limitations: missing energy expenditure data, few participants, no menstrual cycle control, a possible confounding 5-minute warm-up, no matched meal timing, no documentation on room temperature and humidity, and analysis of a limited number of variables.

## Electronic supplementary material

Below is the link to the electronic supplementary material.

Supplementary Material 1

Supplementary Material 2

## Data Availability

The data sets supporting the results reported in the article and the code to reproduce the PCA analysis’ figure is shared via open access. DOI: 10.5281/zenodo.13322184.
