# Supplementary material for: Effects of time-of-day on the noradrenaline, adrenaline, cortisol and blood lipidome response to an ice bath
Source: Sci Rep. 2025 Jan 8;15:1263. doi: 10.1038/s41598-025-85304-8 (PMC11711488; doi:10.1038/s41598-025-85304-8)
Supplement: Supplementary file 1 — Supplementary Material 1 [file 41598_2025_85304_MOESM1_ESM.pdf]

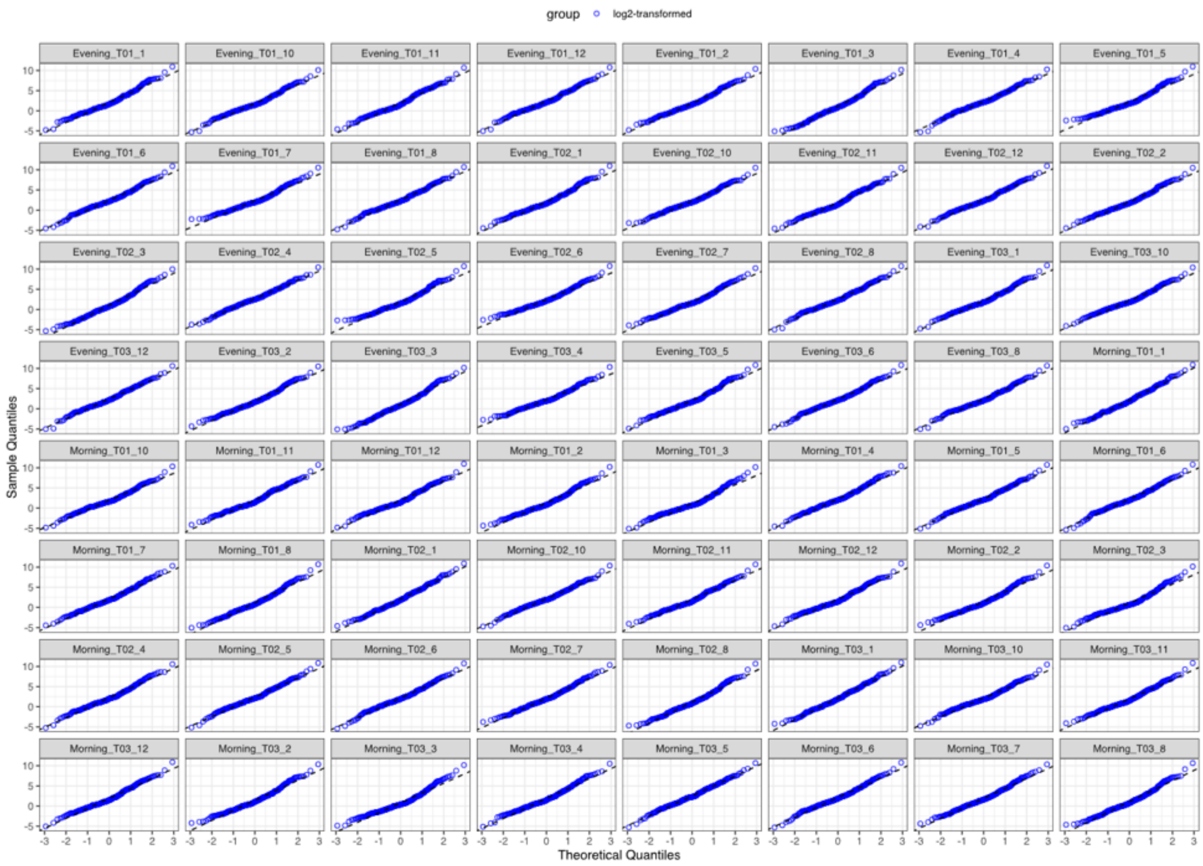

Supplementary material 1 QQ-plots of log2-transformed lipid concentrations. Normal distribution of 11 participants' plasma samples was validated from before (T01), 5 min after (T02) and 30 min after (T03) morning and evening ice baths.
