# Supplementary material for: Effects of time-of-day on the noradrenaline, adrenaline, cortisol and blood lipidome response to an ice bath
Source: Sci Rep. 2025 Jan 8;15:1263. doi: 10.1038/s41598-025-85304-8 (PMC11711488; doi:10.1038/s41598-025-85304-8)
Supplement: Supplementary file 2 — Supplementary Material 2 [file 41598_2025_85304_MOESM2_ESM.pdf]

	Women	Men
**Age (years)**	25 ±6	27 ±6
**BMI (kg/m^2)**	22 ±2	24 ±1
**Weight (kg)**	65 ±5	78 ±8
**Height (cm)**	170 ±3	182 ±6

Supplementary material 2 Participant characteristics of women and men. Age, BMI, body weight and height comparison of women (n = 6) and men (n = 6).
